# Sequential therapy from entecavir to tenofovir alafenamide *versus* continuous entecavir monotherapy for patients with chronic hepatitis B

**DOI:** 10.1002/jgh3.12443

**Published:** 2020-11-02

**Authors:** Norio Itokawa, Masanori Atsukawa, Akihito Tsubota, Koichi Takaguchi, Makoto Nakamuta, Atsushi Hiraoka, Keizo Kato, Hiroshi Abe, Shigeru Mikami, Noritomo Shimada, Makoto Chuma, Nozaki Akito, Haruki Uojima, Chikara Ogawa, Toru Asano, Joji Tani, Asahiro Morishita, Tomonori Senoh, Naoki Yamashita, Tsunekazu Oikawa, Yoshihiro Matsumoto, Mai Koeda, Yuji Yoshida, Tomohide Tanabe, Tomomi Okubo, Taeang Arai, Korenobu Hayama, Ai‐Nakagawa Iwashita, Chisa Kondo, Toshifumi Tada, Hidenori Toyoda, Takashi Kumada, Katsuhiko Iwakiri

**Affiliations:** ^1^ Department of Internal Medicine, Division of Gastroenterology Nippon Medical School Chiba Hokusoh Hospital Chiba Japan; ^2^ Department of Internal Medicine, Division of Gastroenterology and Hepatology Nippon Medical School Tokyo Japan; ^3^ Core Research Facilities The Jikei University School of Medicine Tokyo Japan; ^4^ Department of Hepatology Kagawa Prefectural Central Hospital Takamatsu Japan; ^5^ Department of Gastroenterology National Hospital Organization Kyushu Medical Center Fukuoka Japan; ^6^ Gastroenterology Center Ehime Prefectural Central Hospital Matsuyama Japan; ^7^ Division of Gastroenterology and Hepatology Shinmatusdo Central General Hospital Matsudo Japan; ^8^ Department of Internal Medicine, Division of Gastroenterology Kikkoman General Hospital Noda Japan; ^9^ Department of Internal Medicine, Division of Gastroenterology and Hepatology Otakanomori Hospital Chiba Japan; ^10^ Gastroenterological Center Yokohama City University Medical Center Yokohama Japan; ^11^ Department of Gastroenterology Kitasato University School of Medicine Kanagawa Japan; ^12^ Department of Gastroenterology and Hepatology Takamatsu Red Cross Hospital Takamatsu Japan; ^13^ Department of Internal Medicine, Division of Gastroenterology and Hepatology Tokyo Metropolitan Bokutoh Hospital Tokyo Japan; ^14^ Department of Gastroenterology Kagawa University Graduate School of Medicine Kagawa Japan; ^15^ Department of Gastroenterology and Hepatology The Jikei University School of Medicine Tokyo Japan; ^16^ Department of Gastroenterology and Hepatology Jikei University School of Medicine Kashiwa Hospital Chiba Japan; ^17^ Department of Gastroenterology Ogaki Municipal Hospital Gifu Japan; ^18^ Department of Nursing Ogaki Women's College Gifu Japan

**Keywords:** entecavir, hepatitis B surface antigen, nucleos(*t*)ide analogs, tenofovir alafenamide

## Abstract

**Background and Aim:**

Although tenofovir alafenamide (TAF), as well as entecavir (ETV), is widely used as first‐line treatment for patients with chronic hepatitis B, there are only a few studies comparing sequential therapy from ETV to TAF and continuous ETV monotherapy in patients with maintained virologic response to ETV.

**Methods:**

In a retrospective multicenter study, we investigated the efficacy and safety of sequential therapy from ETV to TAF (ETV‐TAF group) and compared them with continuous ETV monotherapy (ETV group), using propensity score matching, in chronic hepatitis B patients.

**Results:**

From 442 patients, we analyzed 142 patients from each group comprising 71 patients matched for several data, including age, HBV genotype, hepatitis B envelope antigen, cirrhosis, alanine aminotransferase, platelet count, prior ETV monotherapy period, and hepatitis B surface antigen (HBsAg) change during prior ETV monotherapy. In the ETV‐TAF group, HBsAg levels significantly decreased from baseline to 48 weeks after switching to TAF (−0.02 log IU/mL, *P* = 0.038). HBcrAg levels also significantly decreased after switching to TAF (−0.1 log IU/mL, *P* = 0.004). However, there were no significant differences in the reduction of HBsAg and HBcrAg levels between the ETV‐TAF and ETV groups. There was no significant difference in the change of estimated glomerular filtration rate levels from baseline to 48 weeks between the two groups.

**Conclusions:**

The present study indicated that the efficacy, especially of the HBsAg‐reducing action, and safety of sequential therapy from ETV to TAF were similar to those of continuous ETV monotherapy among chronic hepatitis B patients with maintained virologic response to ETV.

## Graphical abstract

In a retrospective, multicenter study involving 17 institutions, we investigated the efficacy and safety of sequential therapy from entecavir (ETV) to tenofovir alafenamide (TAF) (ETV‐TAF group). The present study indicated that the efficacy, especially of the hepatitis B surface antigen‐reducing action, and safety of sequential therapy from ETV to TAF were similar to those of continuous ETV monotherapy.

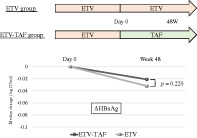

## Introduction

Hepatitis B virus (HBV) infection is one of the most critical infectious diseases, affecting approximately 250 million people worldwide, which comprises 3.5% of the world population.^1^ The chronicity of HBV infection is a major cause of cirrhosis and liver cancer, leading to 780 000 deaths annually.^2^, ^3^ The HBV DNA level is widely recognized as a risk factor for HBV‐related carcinogenesis.^4^ In addition, the level of serum hepatitis B surface antigen (HBsAg) is also closely related to carcinogenesis.^5^ Even though the HBV DNA level is low, the carcinogenic rate remains high in patients with an HBsAg level of ≥3.00 log IU/mL.^6^ Therefore, the ultimate goal of treatment for patients with chronic hepatitis B is to achieve an HBsAg clearance.^7^

Two antiviral drugs, nucleos(*t*)ide analogs (NAs) and interferons (IFNs), are currently available for patients with chronic hepatitis B. IFN shows a prolonged immunoregulatory activity and yields no viral resistance. However, there are some limitations, such as reduced levels of HBV DNA and HBsAg found in limited patients and intolerable adverse effects observed in most patients.^8^, ^9^ In contrast, NAs such as entecavir (ETV) and tenofovir (TFV) strongly suppress HBV replication and rapidly improve HBV viremia and have a high genetic barrier to the development of viral resistance.^10^, ^11^ Given that NAs have fewer adverse effects than those exhibited by IFNs in a clinical setting, they are widely used as first‐line treatment for patients with chronic hepatitis B. However, it is generally considered difficult to eliminate HBV from infected hepatocytes and to reduce the HBsAg levels even in patients receiving long‐term NA therapy, given that NAs do not act on the diminution of covalently closed circular DNA (cccDNA) of HBV.^12^, ^13^

Recently, the HBsAg‐reducing action of tenofovir disoproxil fumarate (TDF) has been reported to be greater than that of ETV in the exploratory analysis of NA‐naïve patients.^14^ Tenofovir alafenamide (TAF), a newly developed prodrug of TFV, is more efficiently incorporated into hepatocytes than TDF and is hydrolyzed to TFV and subsequently phosphorylated to TFV diphosphate.^15^ International phase 3 studies have reported that the reduction of HBsAg levels in TAF was comparable to that in TDF.^16^, ^17^ Although a small sample study of patients with maintained virologic response to ETV has shown similar HBsAg‐reducing effects between continuous ETV monotherapy and sequential therapy from ETV to TDF,^18^ there are few studies comparing continuous ETV monotherapy and sequential therapy from ETV to TAF in patients who had been receiving ETV and who showed maintained virologic response to ETV.

In the present multicenter, retrospective study, we focused on the reduction of HBsAg levels during treatment and investigated the efficacy and safety of sequential therapy from ETV to TAF in patients with chronic hepatitis B compared to continuous ETV monotherapy.

## Methods

### *Subjects*

This multicenter, retrospective study included 442 patients with chronic hepatitis B who were treated with sequential therapy from ETV to TAF or continuous ETV monotherapy at 16 hospitals in Japan. The inclusion criteria were as follows: (i) Patients had been receiving ETV monotherapy for ≥1 year and persistently had serum HBV DNA level of <1.3 log IU/mL for >6 months prior to day 0; (ii) when patients received sequential therapy from ETV to TAF (ETV‐TAF group), the duration of TAF was >1 year after the switching; (iii) when patients received continuous ETV monotherapy without switching to TAF (ETV group), the duration of ETV was >1 year after day 0; (iv) grade 0 or 1 patient status according to the Eastern Cooperative Oncology Group Performance Status criteria; and (v) age ≥ 18 years. The exclusion criteria were as follows: (i) previous treatment with NA other than ETV or combined with other NAs; (ii) presence of resistance to NA; (iii) presence of decompensated cirrhosis or liver failure; (iv) coinfection with hepatitis C virus or human immunodeficiency virus; (v) presence of other chronic liver diseases; (vi) ongoing chemotherapy or immunosuppressor/immnomodulator treatment for any diseases; (vii) presence of severe comorbidities; and (viii) women who were pregnant, expectant mothers, and lactating mothers. This study was designed according to the ethical guidelines of the Helsinki Declaration in 2013 and was approved by the ethics committee of each participating institution. This study was registered in the UMIN Clinical Trials Registry (UMIN 000034362). Written informed consent was obtained from all patients before participation.

### *Study design and treatment protocol*

To compare the ETV‐TAF and ETV groups, we adjusted the baseline factors, including age, HBV genotype, presence/absence of hepatitis B envelope antigen (HBeAg), presence/absence of cirrhosis, serum alanine aminotransferase (ALT) level, platelet count, prior ETV monotherapy period, and HBsAg change for 48 weeks during prior ETV monotherapy, using propensity score (PS) matching. ETV (Baraclude; Bristol‐Myers, Tokyo, Japan) was administered orally at a dosage of 0.5 mg once daily under fasting conditions. TAF (Vemlidy; Gilead Sciences, Tokyo, Japan) was administered orally at a dosage of 25 mg once daily. Day 0 was set as the day when ETV was switched to TAF in the ETV‐TAF group or 48 weeks prior to the latest data in the ETV group.

### *Laboratory tests and* *HBV‐related markers*

Physical, hematological, and biochemical examinations were performed every 3 months during the treatment period. Estimated glomerular filtration rate (eGFR) (mL/min/1.73 mm^2^) was calculated using the following equation generated by the Japanese Society of Nephrology: 194 × (serum creatinine)^−1.094^ × (Age)^−0.287^ (× 0.739 if female).^19^ Serum HBV DNA levels were quantified using Cobas TaqMan HBV v.2.0 (Roche Diagnostics, Tokyo, Japan). Serum HBsAg levels were quantified using the ARCHITECT HBsAg QT assay kit (Abbott Laboratories, Tokyo, Japan). Serum HBV core‐related antigen (HBcrAg) levels were quantified using a fully automated analyzer system (Lumipulse System; Fujirebio, Tokyo, Japan).

### *Treatment endpoints*

The primary end‐point was an HBsAg change from day 0 to 48 weeks after switching to TAF or the corresponding continuous ETV. The changes were compared between the ETV‐TAF and ETV groups. The secondary end‐point was the changes in serum ALT, HBcrAg, and eGFR levels from day 0 to 48 weeks.

### *Statistical analyses*

Continuous variables were presented as medians and ranges, and categorical variables were presented as numbers and percentages. Categorical variables were compared between groups using Fisher's exact test, and continuous variables were analyzed using the Mann–Whitney U test. The kinetics of the aforementioned factors was examined using the Wilcoxon signed‐rank test. PS matching was performed to reduce the differences in baseline characteristics between the ETV‐TAF and ETV groups. PS models were estimated using a logistic regression model that adjusts for patient characteristics between the ETV‐TAF and ETV groups described above. One‐to‐one pairing of patients was completed using nearest‐neighbor matching without replacement. PS was matched using a caliper width of 0.2 logit of the SD. The standardized difference was used to assess the covariate balance, and that of <0.1 suggests adequate variable balance after propensity matching. All statistical analyses were performed using the Excel Statistics 2015 software (SSRI, Tokyo). The level of statistical significance was set at *P* < 0.05.

## Results

### *Patient characteristics*

Of a total of 442 patients, 342 met all the inclusion criteria and none of the exclusion criteria: 113 received sequential therapy from ETV to TAF and 229 continuous ETV monotherapy. The PS matching process for the two treatment groups resulted in a matched sample size that comprised 71 patients in each group, who were subjected to analysis (Table 1). There were no significant differences in the baseline characteristics between the ETV‐TAF and ETV groups.

**Table 1:** Comparison of baseline characteristics between two groups

Factors	ETV‐TAF group (*n* = 71)	ETV group (*n* = 71)	*P* value	Standardized difference
Age (years)^a^	61 (36–86)	58 (34–78)	0.519	0.057
Gender (male/female)	45/26	42/29	0.731	0.067
HBeAg‐positive/HBeAg‐negative	6/65	7/64	1.000	0.014
Noncirrhosis/cirrhosis	58/13	55/16	0.678	0.093
HBV DNA (log IU/mL)^a^	N.D. (N.D.– <1.3+)	N.D. (N.D.– <1.3+)	0.357	0.038
HBsAg (log IU/mL)^a^	2.86 (−0.85 to 4.45)	2.72 (−0.77 to 4.29)	0.142	0.081
HBcrAg (log IU/mL)^a^	3.10 (<2.90–6.70)	3.40 (<2.90–>7.00)	0.207	0.028
HBV genotype (A/B/C/missing)	0/10/46/15	0/11/45/15	1.000	0.015
Platelet count (×10^3^/mm^3^)^a^	17.4 (5.5–45.8)	18.5 (3.1–44.6)	0.991	0.055
Alanine aminotransferase (IU/L)^a^	20 (10–87)	19 (7–118)	0.834	0.046
α‐fetoprotein (ng/mL)^a^	2.8 (1.0–9.0)	2.5 (1.0–18.9)	0.178	0.020
eGFR (mL/min/1.73 m^2^)^a^	72.0 (37.0–124.5)	76.0 (38.0–117.0)	0.141	0.091
Prior ETV monotherapy period (months)^a^	52 (14–175)	57 (12–188)	0.748	0.042
HBsAg change during ETV monotherapy between −48 weeks and day 0 (log IU/mL)^a^	−0.09 (−0.44 to 0.41)	−0.06 (−0.41 to 1.07)	0.669	0.080

^a^ Categorical variables are given as number. Continuous variables are given as median (range).

ETV, entecavir; HBcrAg, hepatitis B virus core‐related antigen; HBeAg, hepatitis B envelope antigen; HBsAg, hepatitis B surface antigen; N.D., not detected; TAF, tenofovir alafenamide.

### *Treatment efficacy in patients treated with sequential therapy from* *ETV* *to* *TAF* *and with continuous* *ETV* *monotherapy*

**Figure 1:**
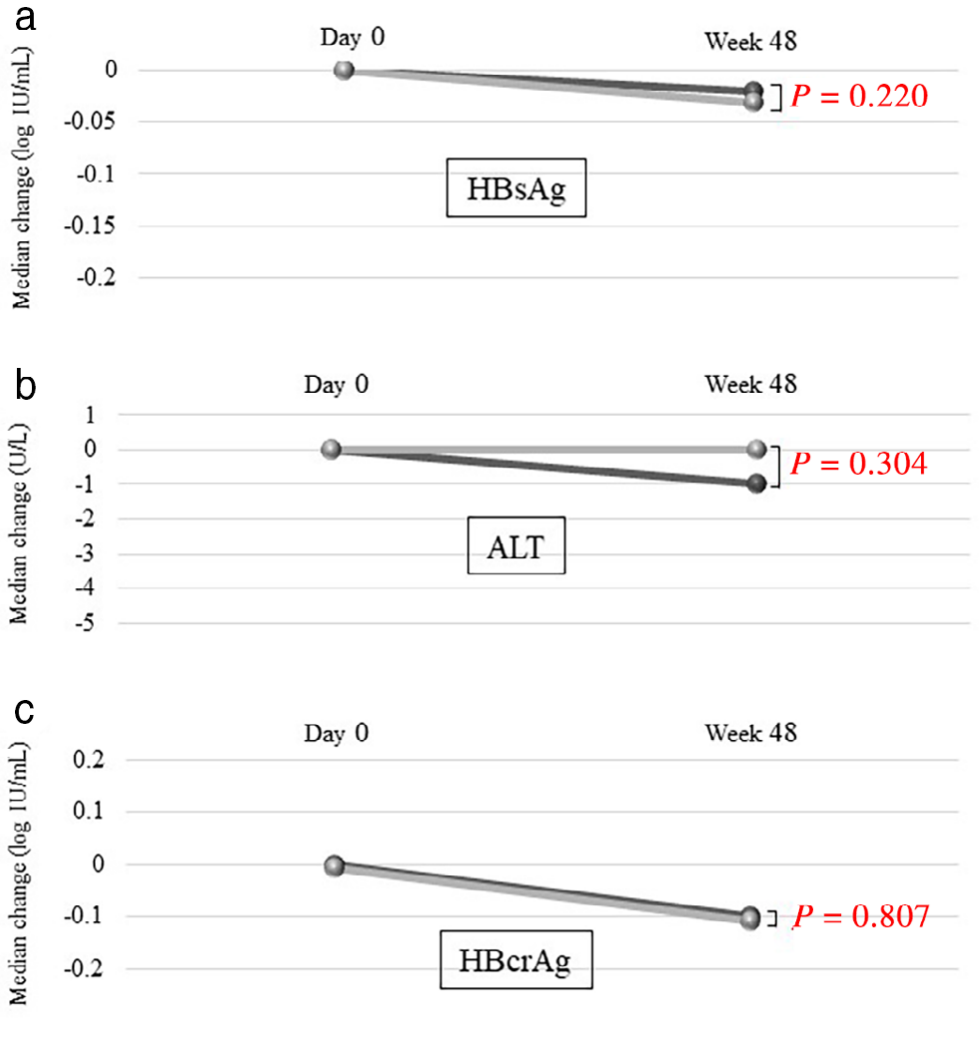
Comparison of treatment efficacies between the ETV‐TAF and ETV groups. The median changes in the (a) HBsAg levels: (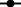), ETV‐TAF; (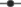), ETV; (b) ALT levels: (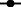), ETV‐TAF; (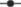), ETV, and (c) HBcrAg levels: (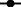), ETV‐TAF; (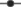), ETV. ALT, alanine aminotransferase; ETV, entecavir; HBcrAg, hepatitis B virus core‐related antigen; HBsAg, hepatitis B surface antigen; TAF, tenofovir alafenamide.

In the ETV‐TAF group, the median changes in HBsAg, ALT, and HBcrAg levels from day 0 to 48 weeks were −0.02 log IU/mL (*P* = 0.038), −1 U/L (*P* = 0.258), and −0.1 log IU/mL (*P* = 0.004), respectively (Fig. 1). In the ETV group, they were −0.03 log IU/mL (*P* < 0.001), 0 U/L (*P* = 0.902), and − 0.1 log IU/mL (*P* = 0.006), respectively (Fig. 1). Thus, HBsAg and HBcrAg (but not ALT) levels significantly decreased from day 0 to 48 weeks in both the ETV‐TAF and ETV groups. However, there were no significant differences in these virologic and biological changes between the ETV‐TAF and ETV groups (*P* = 0.220 for HBsAg, Fig. 1a; 0.304 for ALT, Figure 1b; and 0.807 for HBcrAg, Figure 1c). There were no significant differences in the rates of patients with serum ALT normalization (<30 IU/L) at 48 weeks between the ETV‐TAF and ETV groups (87.3% [62/71] and 83.1% [59/71], respectively; *P* = 0.637).

### *Comparison of* *HBsAg* *reduction between the* *ETV‐TAF* *and* *ETV* *groups based on the baseline factors*

Patients in each treatment group were subdivided into two subcategories based on the presence/absence of HBeAg, HBV genotype, and cutoff value of the baseline HBsAg level.

In the ETV‐TAF group, the median HBsAg changes from day 0 to 48 weeks in the subgroups with baseline HBsAg level <3.00 log IU/mL and ≥3.00 log IU/mL were −0.02 log IU/mL (*P* = 0.096) and −0.02 log IU/mL (*P* = 0.131), respectively. The median HBsAg changes in the HBeAg‐positive and HBeAg‐negative subgroups were −0.01 log IU/mL (*P* = 0.753) and −0.02 log IU/mL (*P* = 0.039), respectively. The median HBsAg changes in the HBV genotype B and C subgroups were −0.06 log IU/mL (*P* = 0.185) and 0 log IU/mL (*P* = 0.245), respectively.

In the ETV group, the median HBsAg changes from day 0 to 48 weeks in the subgroups with baseline HBsAg levels <3.00 log IU/mL and ≥3.00 log IU/mL were −0.03 log IU/mL (*P* = 0.003) and −0.03 log IU/mL (*P* = 0.025), respectively. The median HBsAg changes in the HBeAg‐positive and HBeAg‐negative subgroups were −0.02 log IU/mL (*P* = 0.933) and −0.05 log IU/mL (*P* < 0.001), respectively. The median HBsAg changes in the HBV genotype B and C subgroups were −0.02 log IU/mL (*P* = 0.450) and −0.03 log IU/mL (*P* < 0.001), respectively.

Collectively, in both the ETV‐TAF and ETV groups, the HBsAg levels significantly decreased from day 0 to 48 weeks in the subgroups with baseline HBsAg levels <3.00 log IU/mL and negative HBeAg. In contrast, the HBsAg levels did not decrease or decreased less significantly in the subgroups with baseline HBsAg levels ≥3.00 log IU/mL and positive HBeAg.

**Figure 2:**
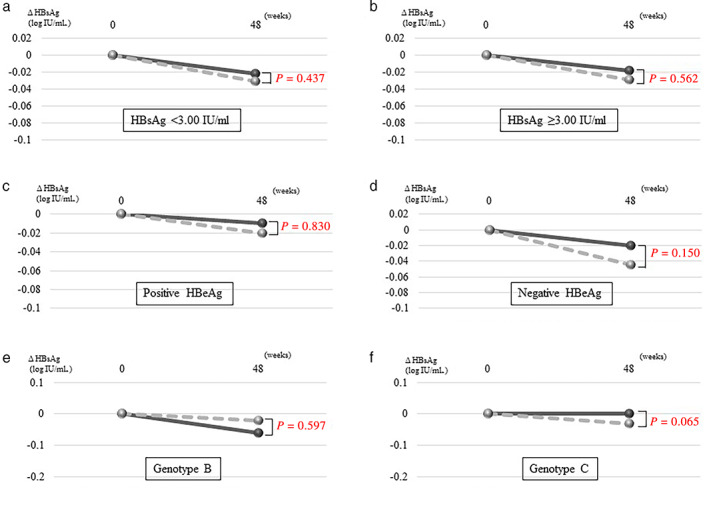
Comparison of HBsAg reduction between the two subgroups according to the baseline factors in the ETV‐TAF and ETV groups. The median HBsAg changes in the subgroups with (a) HBsAg <3.00 log IU/mL: (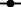), ETV‐TAF: HBsAg < 3.00 log IU/mL; (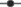), ETV: HBsAg < 3.00 log IU/mL, (b) HBsAg ≥3.00 log IU/mL: (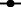), ETV‐TAF: HBsAg ≥ 3.00 log IU/mL; (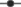), ETV: HBsAg ≥ 3.00 log IU/mL, (c) positive HBeAg: (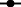), ETV‐TAF: HBeAg positive; (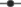), ETV: HBeAg positive, (d) negative HBeAg: (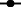), ETV‐TAF: HBeAg negative; (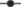), ETV: HBeAg negative, (e) HBV genotype B: (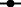), ETV‐TAF: genotype B; (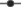), ETV: genotype B, and (f) HBV genotype C: (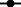), ETV‐TAF: genotype C; (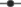), ETV: genotype C. ETV, entecavir; HBeAg, hepatitis B envelope antigen; HBsAg, hepatitis B surface antigen; TAF, tenofovir alafenamide.

The reduction in HBsAg was compared between the ETV‐TAF and ETV groups in each categorical subgroup. Regardless of the baseline HBsAg level (Fig. 2a,b), HBeAg status (Fig. 2c,d), and HBV genotype (Fig. 2e,f), the differences in the HBsAg reduction between the two treatment groups were not statistically significant.

### *Safety*

**Figure 3:**
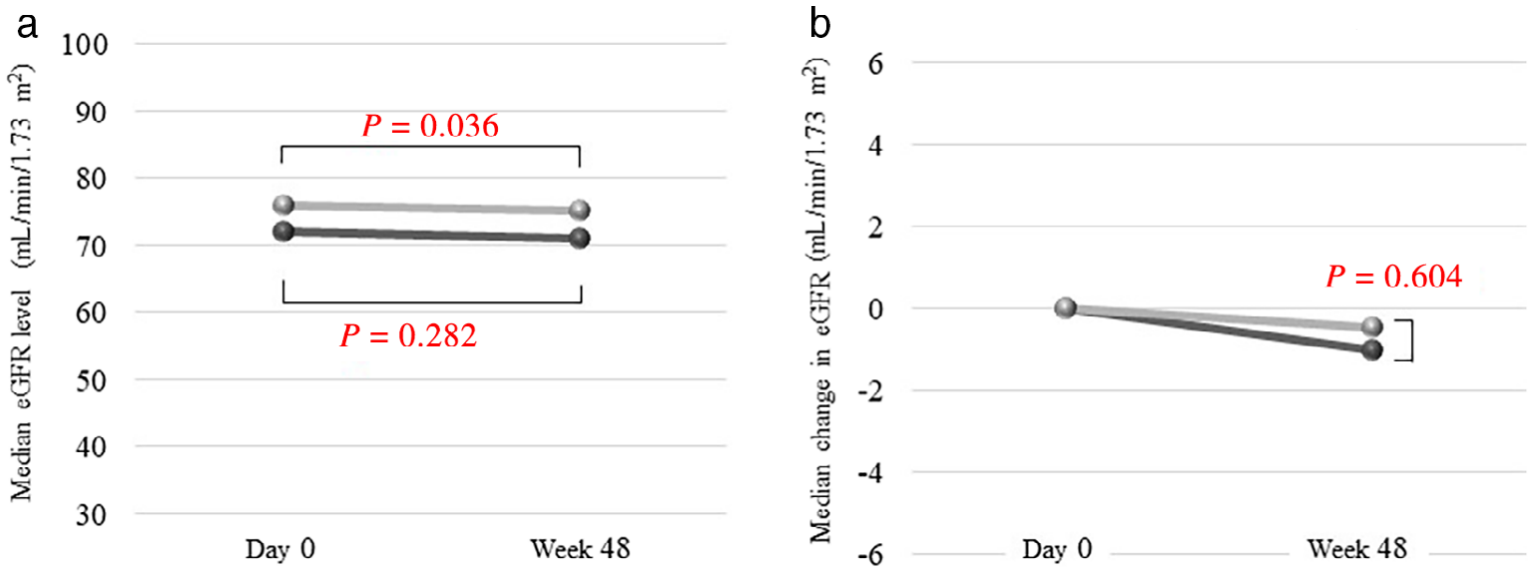
Changes in eGFR level and comparison between the two groups. (a) Median eGFR levels in the ETV‐TAF and ETV groups: (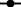), ETV‐TAF; (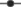), ETV. (b) Median changes in the eGFR levels in the ETV‐TAF and ETV groups: (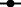), ETV‐TAF; (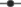), ETV. eGFR, estimated glomerular filtration rate.

The median eGFR levels at day 0 and 48 weeks in the ETV‐TAF and ETV groups are shown in Figure 3a. In the ETV‐TAF group, the median eGFR level significantly decreased after switching to TAF (from 72.0 mL/min/1.73 mm^2^ to 70.9 mL/min/1.73 mm^2^, *P* = 0.036). It also decreased in the ETV group, although the difference was not statistically significant (from 76.0 mL/min/1.73 mm^2^ to 75.3 mL/min/1.73 mm^2^, *P* = 0.282). Although the decline of eGFR in the ETV‐TAF group was significant, but not in the ETV group, there were no significant differences in the eGFR changes between the ETV‐TAF and ETV groups (−1.0 mL/min/1.73 mm^2^ *vs* −0.5 mL/min/1.73 mm^2^, *P* = 0.604; Figure 3b).

## Discussion

The present study assessed the efficacy and safety of sequential therapy from ETV to TAF and compared them with those of continuous ETV monotherapy, using PS matching, in Japanese patients with chronic hepatitis B. We specifically focused on the HBsAg reduction in the two treatment groups, which led to the following findings: (i) HBsAg reduction in patients receiving sequential therapy was similar to that in patients receiving continuous ETV monotherapy; (ii) both the treatment regimens significantly reduced the HBsAg levels, especially in the subgroups with baseline HBsAg level <3.00 log IU/mL and negative HBeAg; and (iii) there was no significant difference in the eGFR changes between the two treatment groups.

NAs (such as ETV and prodrugs of TFV) are widely used as first‐line antiviral therapy for patients with HBV in clinical settings because of their potent antiviral activities against HBV and high genetic barrier to drug resistance.^10^, ^11^, ^20^ In a randomized prospective comparative study between ETV and TDF, the HBsAg reduction in patients treated with TDF was greater than that in patients treated with ETV.^14^ International phase 3 studies showed a comparable HBsAg‐reducing action between TDF and TAF.^16^, ^17^ Many physicians assume that switching from ETV to TAF may be beneficial for HBsAg reduction based on the results of these previous studies, which included many NA‐naïve patients but not those who had been receiving ETV and exhibiting maintained virologic response.

Regarding HBsAg reduction, a few studies have addressed the comparison between sequential therapy from ETV to TFV prodrug and continuous ETV monotherapy.^18^, ^21^ A randomized controlled trial switched patients on long‐term ETV treatment to TDF and continuous ETV and found no significant differences in the HBsAg reduction between the two groups.^18^ Meanwhile, a prospective comparative study of patients with virologic response to ETV suggested that sequential therapy to TAF may have a greater HBsAg‐reducing effect than continuous ETV among patients with low baseline HBsAg levels.^21^ However, the present study demonstrated that the HBsAg reduction in the ETV‐TAF group was similar to that in the ETV group. We also compared the HBsAg changes according to the baseline HBsAg levels; consequently, there were no significant differences in the HBsAg reduction between the two treatment groups in patients with baseline HBsAg levels <3.00 log IU/mL (*n* = 41) or ≥3.00 log IU/mL (*n* = 30). Given that only a few patients with low baseline HBsAg levels (<800 IU/mL; *n* = 6) were included in the aforementioned study,^21^ the cohort size might have caused the difference in results.

Concerning other factors associated with HBsAg reduction, previous studies of NA‐naïve patients receiving TFV treatment showed a greater HBsAg decline in HBeAg‐positive patients than in HBeAg‐negative patients.^14^, ^16^, ^17^ In contrast, a comparative study between switching to TAF and continuous ETV suggested that there was no significant difference in the HBsAg‐reducing effect, regardless of the presence/absence of HBeAg.^21^ In the present study, the HBsAg levels significantly decreased in the HBeAg‐negative (but not HBeAg‐positive) subgroups in both treatment groups, and there were no significant differences between the treatment groups, irrespective of the presence/absence of HBeAg. A previous study based on sequential therapy to TAF reported that the degree of HBsAg reduction was higher in patients with genotype B.^22^ In fact, the present study showed that HBsAg reduction in the ETV‐TAF group was significant in patients with genotype B, unlike in those with genotype C. However, there were no significant differences between ETV‐TAF and ETV groups in each HBV genotype.

A previous study has reported that serum HBcrAg, unlike HBsAg, is neither transcribed nor translated from the integrated HBV sequence, and therefore reflects the intracellular levels of HBV cccDNA, and that HBcrAg levels change independent of changes in the HBV DNA level during NA administration.^23^ Therefore, as described in the present study, it is conceivable that a notable HBcrAg‐reducing effect was not observed even when switching to TAF, despite potent HBV suppression. However, it is noteworthy that both sequential therapy to TAF and continuous ETV monotherapy significantly reduced the HBcrAg levels.

Nephrotoxicity is one of the major adverse events common to treatment with TFV prodrugs.^24^, ^25^ TFV is excreted from the kidney via the proximal tubules. High concentrations of TFV in the tubular epithelial cells inhibit mitochondrial polymerase γ and cause mitochondrial disorders, and thus may cause kidney damage.^26^ Although both TAF and TDF are prodrugs of TFV, international phase 3 studies have reported that the incidence of TFV‐related kidney damage was significantly lower in patients treated with TAF than in those treated with TDF because plasma concentrations of TFV were lower in TAF‐treated patients.^16^, ^17^ ETV is also excreted from the kidneys,^27^ while ETV is generally considered to not affect the renal function in a clinical setting.^28^ In addition, a previous study has reported that eGFR was restored with time in patients with chronic kidney disease who received ETV,^29^ although the reason and mechanism remain unclear. In the present study, no significant difference was observed in the eGFR changes between patients receiving sequential therapy to TAF and those receiving continuous ETV monotherapy. Furthermore, these changes in the renal function were similar to the age‐related changes in the Japanese general population.^30^

Initially, this study was conducted in anticipation of benefits from switching to TAF in terms of the HBsAg reduction. However, the present study failed to demonstrate superiority in the HBsAg‐reducing effect of sequential therapy to TAF compared to that of continuous ETV monotherapy. Furthermore, the treatment efficacy on the ALT and HBcrAg levels, and safety in patients receiving sequential therapy to TAF, was comparable to that in patients receiving continuous ETV monotherapy. Considering the benefit of switching from ETV to TAF, the aspects of medication adherence may be conceivable. ETV must be administered under fasting conditions, while TAF can be administered at any time, once a day. Adherence to NA therapy is crucial to improve the prognosis of chronic hepatitis B. Recent studies have reported the improvement of adherence to medications by switching from ETV to TAF.^22^, ^31^

The present study had some limitations. First, although the baseline characteristics between the two groups were adjusted using PS matching, this study was neither prospective nor randomized. Second, the follow‐up duration was short and, therefore, needs to be extended in the future to clarify the long‐term effects, including the inhibitory action of sequential therapy on carcinogenesis. Third, in the present study with the short follow‐up period, the sample size of patients analyzed was too small to investigate the difference in the HBsAg reduction between the two treatment groups.

Finally, regarding adverse events, the present study did not assess the changes in serum phosphorus levels, bone mineral density, or markers for a renal tubular function including proteinuria.

In conclusion, the present study suggests that there are no significant differences in the efficacy, especially on HBsAg reduction, and safety between sequential therapy from ETV to TAF and continuous ETV monotherapy among chronic hepatitis B patients with maintained virologic response to ETV.
